# Pathogenicity comparison of duck Tembusu virus in different aged Cherry Valley breeding ducks

**DOI:** 10.1186/s12917-019-2020-8

**Published:** 2019-08-06

**Authors:** Chuanwei Lv, Rong Li, Xingpo Liu, Ning Li, Sidang Liu

**Affiliations:** 1 College of Animal Science and Technology, Shandong Agricultural University, 61 Daizong Street, Taian City, 271018 Shandong Province China; 2 Shandong Provincial Key Laboratory of Animal Biotechnology and Disease Control and Prevention, Shandong Agricultural University, 61 Daizong Street, Taian City, 271018 Shandong Province China; 3 Shandong Provincial Engineering Technology Research Center of Animal Disease Control and Prevention, Shandong Agricultural University, 61 Daizong Street, Taian City, 271018 Shandong Province China

**Keywords:** Duck Tembusu virus, Breeding duck, Age, Pathogenicity

## Abstract

**Background:**

Although several studies have revealed that the sensitivity of ducklings to duck Tembusu virus (DTMUV) was related to age, however, DTMUV was originally isolated from egg-laying ducks, and the ovary was the target organ of this virus. Cherry Valley breeding ducks aged 15- and 55-week-old (they are reserve breeding ducks and the normal egg-laying breeding ducks, respectively) were infected with DTMUV, using intramuscular injection, to study the effect of age-related difference on the pathogenicity of DTMUV in breeding ducks.

**Results:**

Examinations of clinical symptoms, gross and microscopic lesions, viral loads, cytokines and serum neutralizing antibodies were performed. Results showed that obvious clinical symptoms, such as depression, ruffled feathers, ataxia and egg-laying drop were observed in the 55-week-old laying ducks, with five ducks dying at 5–7 days post infection (dpi). The 15-week-old ducks showed slight symptoms during infection. Gross lesions were severe and characterized by the congestion, hemorrhage and swelling of some organs in the 55-week-old ducks, including the hemorrhage of endocardium, hepatomegaly, splenomegaly, oviduct hemorrhage, hyperemia and deformation of the ovary. Mild endocardial hemorrhage and hepatosplenomegaly were observed in the 15-week-old ducks. Similarly, there was a significant difference in microscopic lesions between the two groups. The older ducks displayed severe microscopic lesions, specifically in the hemorrhage, interstitial inflammatory cell infiltration of the endocardium, typical viral encephalitis and hemorrhage in the ovary. But on the whole, the 15-week-old ducks showed milder lesions. Viral loads in tissues of the older group were significantly higher than those of the younger group. The levels of interferon (IFN)-γ, interleukin (IL)-2 and neutralizing antibody in the 15-week-old ducks were higher than in the 55-week-old ducks at the early stage of the DTMUV infection, suggesting the immune response in the younger ducks to DTMUV was stronger than in the older ducks.

**Conclusions:**

These results demonstrated that age-related differences in susceptibility to DTMUV in breeding ducks was significant, with 55-week-old egg-laying ducks being more susceptible to DTMUV than 15-week-old reserve breeding ducks.

## Background

In April 2010, an outbreak of infectious duck disease was reported in south-eastern China, characterized by a severe egg production decline in egg-laying ducks [1]. This disease spread rapidly to the main duck-producing regions of China, including Jiangsu, Zhejiang, Fujian, Anhui and Shandong [2, 3], and could also infect chickens, geese and house sparrows [4–6]. The morbidity rate of the ducks in these areas was almost as high as 100% and mortality varied from 5 to 30%, with the deaths potentially resulting from secondary bacterial infections. The disease, causing great economic loss to the duck industry, was first named duck hemorrhagic ovaritis and further study confirmed the causative agent was duck Tembusu virus (DTMUV) [3].

DTMUV is a member of the Flavivirus genus, the Flaviviridae family, and possesses a single-stranded, positive-sense RNA genome [7]. To date, steady research progress into this disease has been made based on the access to complete genome of DTMUV, such as by serological, etiological detection methods [8–10], epidemiological investigation [11], and the modes and vectors of transmission [3, 4, 12–14]. Recently, the interaction between host immune response and DTMUV infection has been a research focus. Some reports showed that DTMUV could trigger the innate immune responses via the Melanoma differentiation-associated protein 5 and Toll-like receptor 3 signaling pathway [15, 16], Sun et al. [17] studied the mammalian host cell responses to DTMUV infection and screened some antiviral proteins through the iTRAQ method in DTMUV-infected BHK-21 cell.

Many research studies have shown that the host age had an important impact on the pathogenicity of some viruses such as avian influenza virus, West Nile virus and Japanese encephalitis virus [18–20]. Moreover, several studies have revealed that the sensitivity of ducklings to DTMUV was related to age: the younger the ducklings were, the more severe pathogenicity the DTMUV had [21, 22]. However, DTMUV was originally isolated from egg-laying ducks, and the ovary was the target organ of this virus. Given that the 55-week-old ducks are raised as the normal egg-laying breeding ducks clinically and the 15-week-old ducks are the reserve breeding ducks, thereby these two kinds of ducks were selected in the present study to investigate if there was any age-related difference in susceptibility to DTMUV in breeding ducks compared with age-matched control groups.

## Results

### Clinical signs and gross lesions

**Fig. 1:**
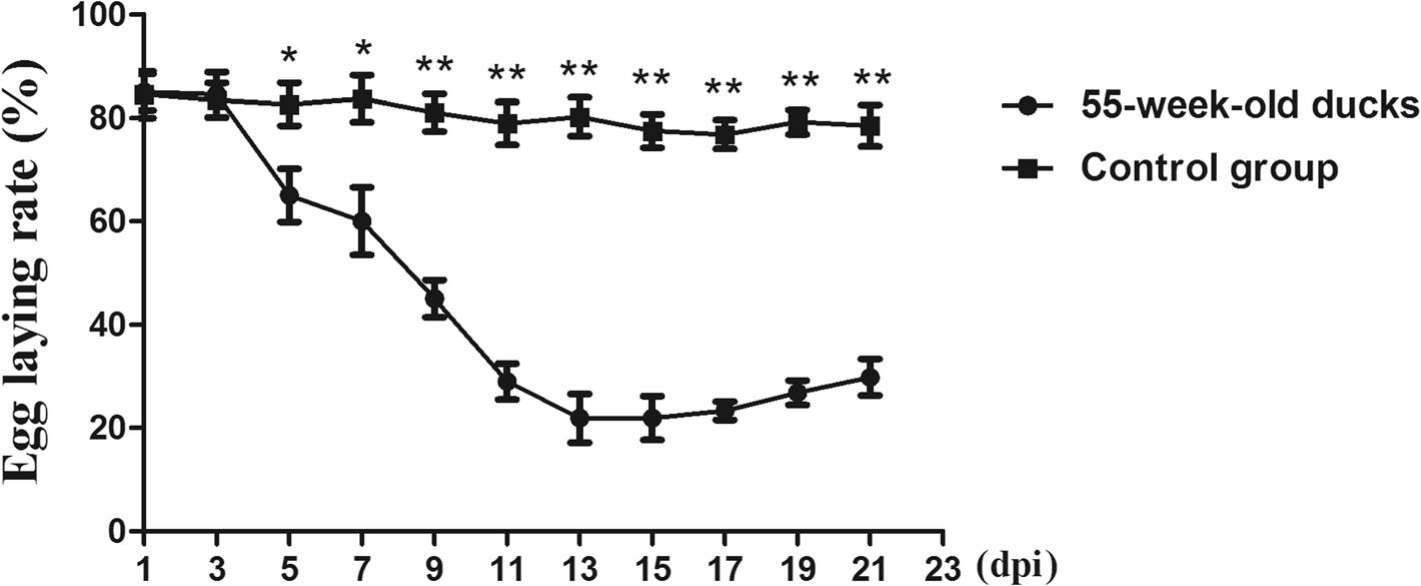
The kinetics of egg production in 55-week-old ducks. Vertical axis indicates the egg-laying rate and horizontal axis indicates the days post infection. Data were shown as means ± SD (*n* = 3). The statistical differences between DTMUV-infected 55-week-old ducks and the age-matched control group were evaluated by Student’s t-test. * *p <* 0.05, ** *p <* 0.01

Throughout the experiment, there were no clinical symptoms and gross lesions in either control group. However, varying degrees of clinical signs and lesions were observed in 15- and 55-week-old breeding ducks, especially in the older ducks. At 3 days post infection (dpi), about 40% of the challenged 55-week-old ducks displayed depression and inappetence, while some excreted white loose stool. At 5–9 dpi, five inoculated ducks died, and another eight ducks showed neurological signs, characterized by dystaxia and paralysis. The feed intake of 55-week-old ducks started to increase and the symptoms gradually disappeared after 11 dpi. In addition, the normal egg production rate of 55-week-old ducks was about 83%, but it was found that the egg production rate of DTMUV-infected ducks began to decline significantly at 5 dpi, and continued until 21 dpi, with a lowest value (21.86%) at 13 dpi (Fig. 1). By comparison, the transient symptoms of DTMUV-infected 15-week-old ducks, such as depression and loss of appetite, were observed at 3–5 dpi, but the symptoms gradually disappeared after 9 dpi and no deaths occurred during the infection.

**Fig. 2:**
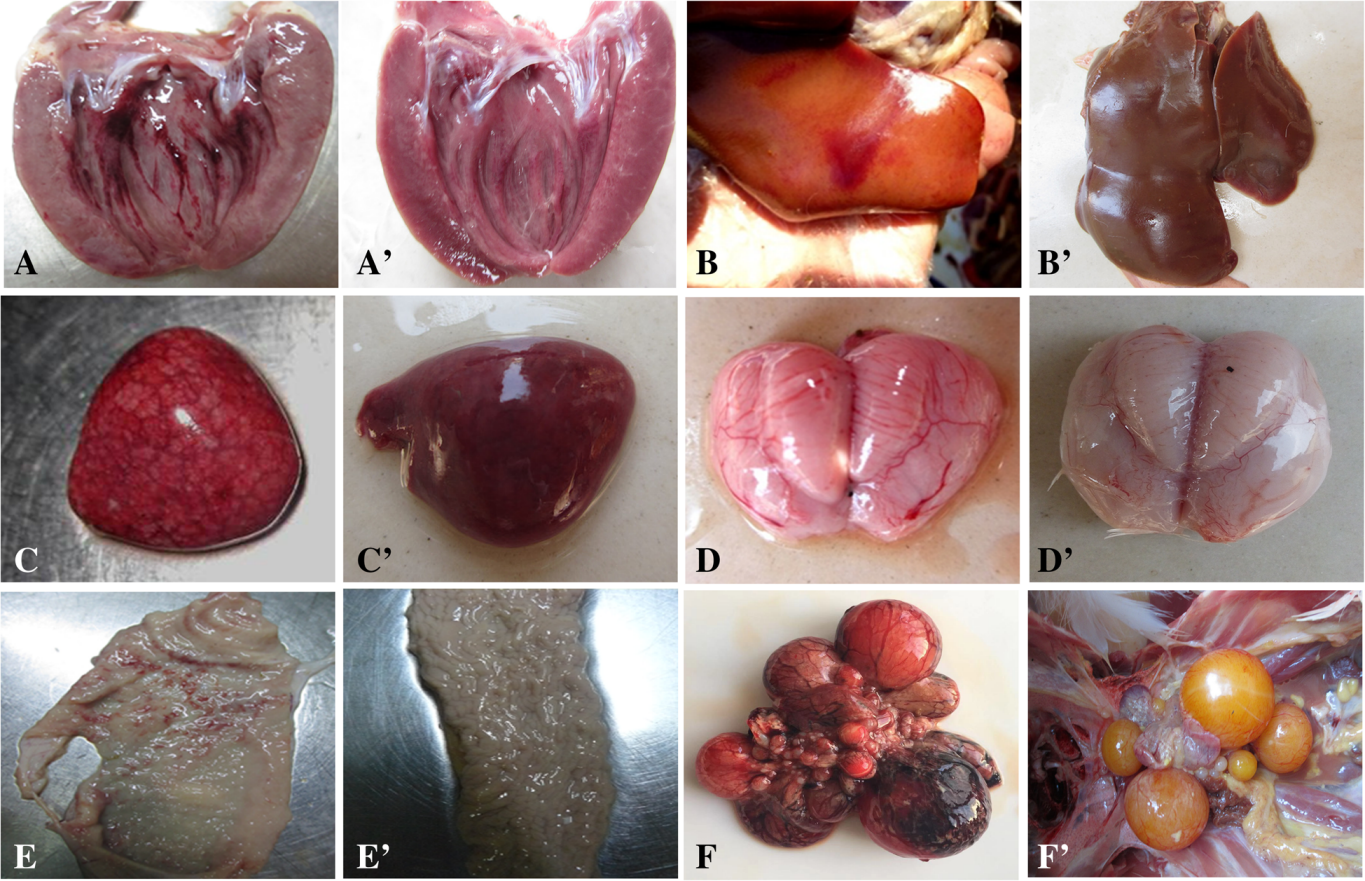
Gross lesions of 55-week-old ducks. **a** Hemorrhage of endocardium, at 5 dpi. **b** Enlarged liver with blood spots, at 5 dpi. **c** Splenomegaly with surface mottling, at 5 dpi. **d** Congested meninx, at 7 dpi. **e** Oviduct with hemorrhage, at 7 dpi. **f** Deformation and necrosis of ovary, at 5 dpi. **a**’, **b**′, **c**′, **d**’, **e**’, and **f**′ were the corresponding normal organs in control group

As regards gross lesions, the 55-week-old ducks also showed more severe lesions characterized by congestion, hemorrhage and swelling of some organs. The hemorrhage of endocardium was the most prevalent lesion (Fig. 2a), while others were hepatomegaly with hemorrhage dots (Fig. 2b), splenomegaly with mottled surface (Fig. 2c), leptomeninges congestion (Fig. 2d), hemorrhage dots of oviducts (Fig. 2e), and ovary hyperemia and deformation (Fig. 2f). The gross lesions of the 15-week-old ducks mainly occurred on the endocardial hemorrhage and hepatosplenomegaly, but the lesions were mild. And there was no lesions in the control group (Fig. 2a’, b’, c’, d’, e’, and f’).

### Microscopic lesions

**Fig. 3:**
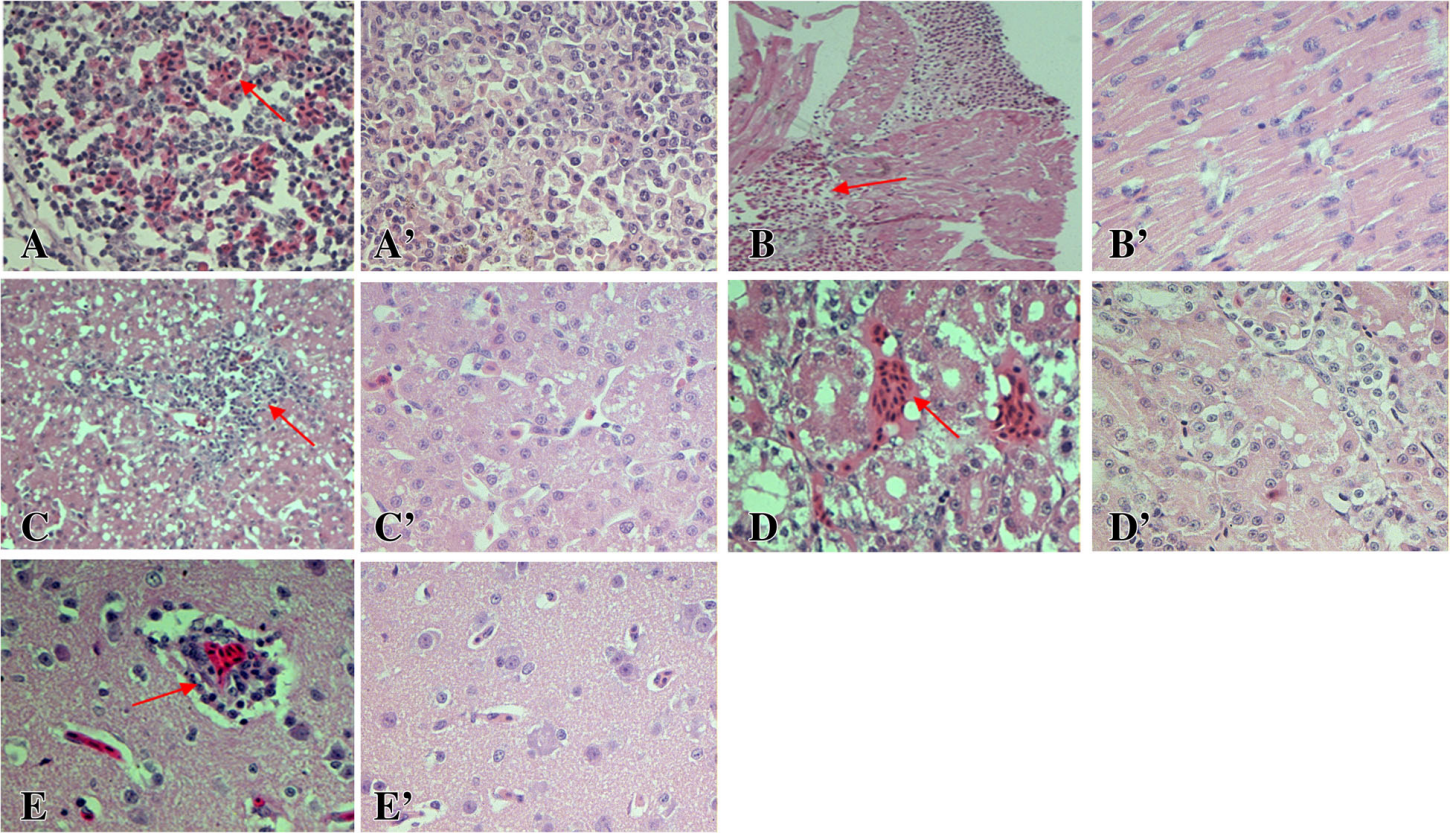
Histopathological changes of 55-week-old ducks. **a** Heterophilic granulocyte infiltration and lymphocyte reduction, spleen (400×). **b** Granular degeneration and necrosis of cell with hemorrhage, heart (200×). **c** Vacuolated degeneration and inflammatory cell infiltration, liver (200×). **d** Granular degeneration and interstitial hemorrhage, kidney (400×). **e** Vascular cuffing, brain (400×). **a**’, **b**′, **c**′, **d**’, and **e**’ were the normal spleen, heart, liver, kidney and brain in control group, and the magnification is 400×

According to the histopathological analysis, the microscopic lesions mainly consisted of alterative inflammation and hemorrhage of parenchymal organ (heart, liver, spleen, kidney, and brain) and interstitial proliferation with inflammatory cell infiltration. Similar to the results of the gross lesions, varying degrees of microscopic lesions were observed in both 15- and 55-week-old ducks, with more severe lesions in the older ducks (Table 1), and with the lesions gradually decreasing after 9 dpi. No obvious microscopic lesions were found in the age-matched control groups (Fig. 3a’, b’, c’, d’, and e’).

**Table 1:** The scoring of microscopic lesions of DTMUV-infected ducks at 5 dpi

Groups	15-week old ducks					55-week old ducks				
Score	0	1	2	3	4	0	1	2	3	4
Organs										
Heart^*^		2/3 ^a^	1/3					1/3	2/3	
Liver		3/3					1/3	2/3		
Spleen^*^		1/3	2/3							3/3
Kidney			3/3						2/3	1/3
Brain^*^		2/3	1/3						3/3	
Ovary^*^		1/3	2/3						1/3	2/3

Annotation: a Number with lesions of the three chickens examined. The severity of microscopic lesions on the organs of the infected ducks were scored from 0 to 4 as follows: 0, normal; 1, the range of the lesions (cell degeneration, necrosis, hemorrhage, inflammatory cell infiltration, etc.) was less than 10%; 2, the range of the lesions was between 10 and 30%; 3, the range was between 30 and 50%; 4, the range was more than 50%. * *p <* 0.05

In the spleen, disruption and necrosis of lymphocytes was found, with the number of lymphocytes in the white pulp significantly decreased with hemorrhage and heterophilic granulocyte infiltration in the 55-week-old ducks (Fig. 3a). The 15-week-old ducks displayed mild necrosis of lymphocytes with heterophilic granulocyte infiltration. In the heart, the 55-week-old ducks showed severe degeneration, necrosis and myocardial hemorrhage with macrophage infiltration (Fig. 3b). However, the 15-week-old ducks demonstrated granular degeneration of myocardial fibers and scattered hemorrhage.

In the liver, severe vacuolated degeneration and necrosis of hepatocytes and inflammatory cell infiltration were observed in the 55-week-old ducks (Fig. 3c). The 15-week-old ducks showed slight vacuolated degeneration and heterophilic granulocyte. In the kidney, granular degeneration and necrosis had occurred on the epithelia of the renal tubules, as well as interstitial hemorrhage in the 55-week-old ducks (Fig. 3d). Only granular degeneration and slight necrosis were observed in the epithelia of the renal tubules in the 15-week-old ducks.

In the brain, vascular congestion, endothelial cell swelling and degeneration were observed in the 55-week-old ducks. Subsequently, typical viral encephalitis was found, including the phenomenon of “vascular cuff”, proliferation of glial cells (Fig. 3e). The younger ducks displayed widened gaps around small blood vessels and moderate proliferation of glial cells.

### Viral loads in tissues

**Fig. 4:**
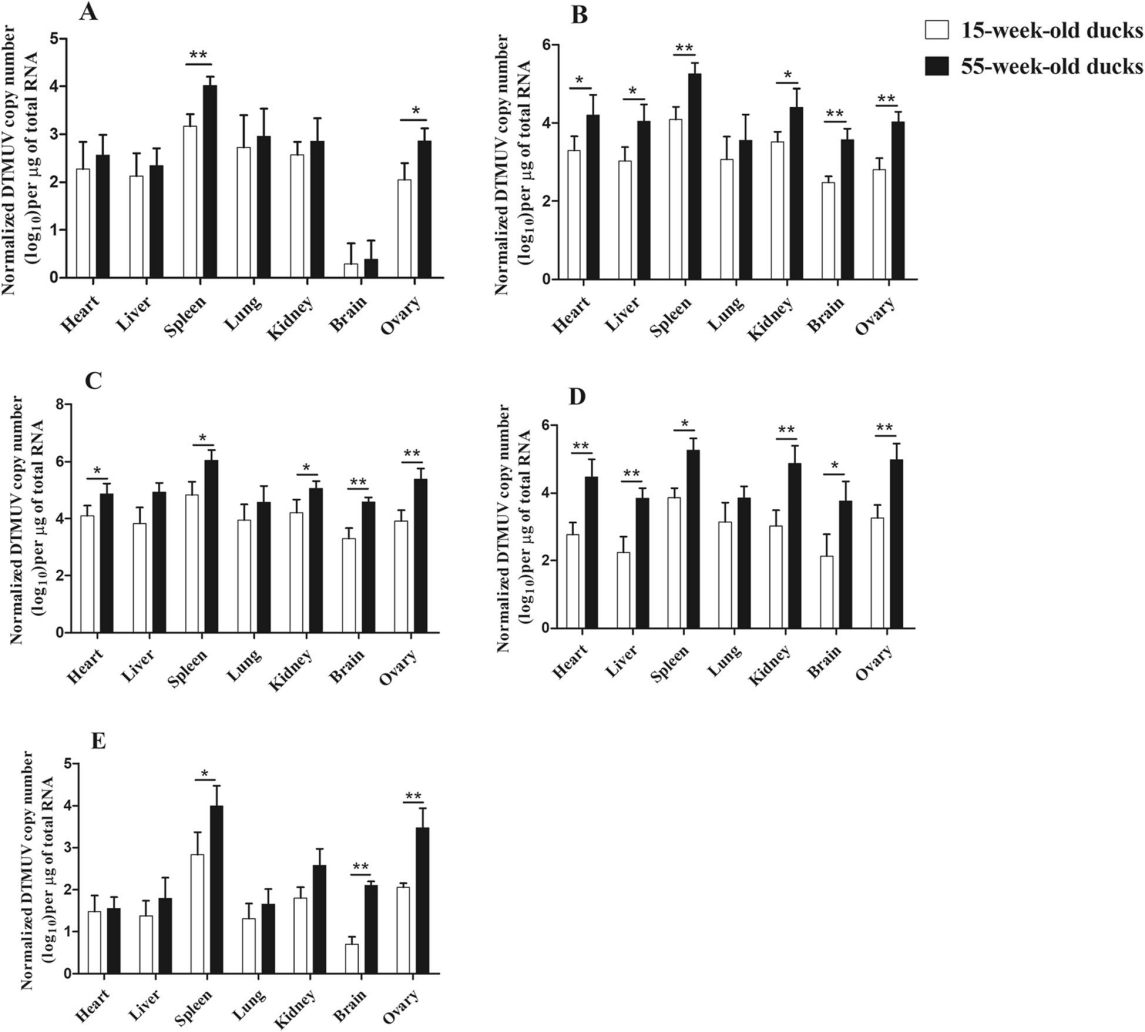
Virus copies in different tissues of DTMUV-infected ducks. **a**, **b**, **c**, **d**, and **e** show the virus copies in heart, liver, spleen, lung, kidney, brain, and ovary of the 15- and 55-week-old ducks at 1, 3, 5, 7 and 9 dpi, respectively. Data were expressed as mean ± SD (*n* = 3). The statistical differences between both groups were evaluated by Student’s t-test. * *p <* 0.05, ** *p <* 0.01

The quantitative real-time PCR (qPCR) was used to determine virus loads in the different tissues of DTMUV-infected ducks. No positive viral RNA was found in any of the control ducks. As can be seen in Fig. 4a, DTMUV could be detected in all tested tissues in both experimental groups at 1 dpi. Notably, the highest number of copies was observed in the spleen, with 1.0 × 10^3.2^ copies/μg (15-week-old ducks) and 1.0 × 10^4.0^ copies/μg (55-week-old ducks), respectively (*p <* 0.01). In the ovary, viral loads also reached a high value, and there was a significant difference between the two groups (*p <* 0.05). At 3 dpi, virus loads increased substantially in all tissues, and copies in the ovary reached 1.0 × 10^4.0^ copies/μg in the 55-week-old ducks (*p <* 0.01, Fig. 4b). The virus loads in the heart, spleen, kidney and brain of the older ducks were significantly higher than in the younger ducks (*p <* 0.01 or *p <* 0.05, Fig. 4b). At 5 dpi, virus loads in most tissues of both groups peaked, such as the 1.0 × 10^4.6^ copies/μg and 1.0 × 10^5.4^ copies/μg detected in the brain and ovary respectively, of the 55-week-old ducks (Fig. 4c). Significant differences were observed in the heart, spleen, kidney, brain and ovary of both groups at 5 dpi (*p <* 0.01 or *p <* 0.05, Fig. 4c). At 7 dpi, viral loads of the tested tissues demonstrated a decreasing trend, but copies in the 55-week-old ducks were significantly higher than in the 15-week-old ducks, except for the lung (Fig. 4d). At 9 dpi, viral loads continued to decrease, but they still remained relatively high in the spleen, kidney, brain, and ovary, with significant differences (*p <* 0.01 or *p <* 0.05, Fig. 4e).

### Interferon (IFN)-γ and interleukin (IL)-2 level in serum

**Fig. 5:**
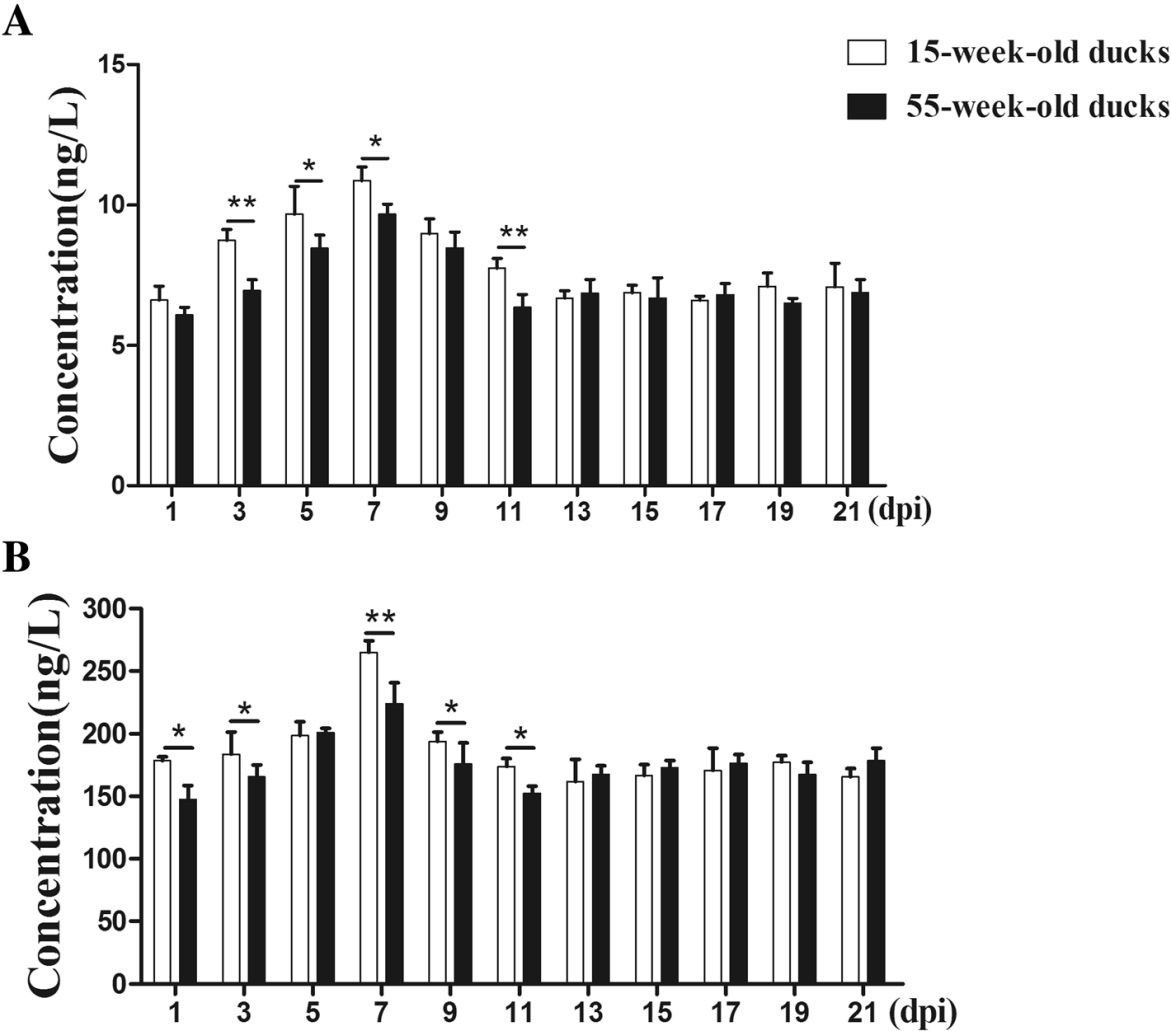
IFN-γ and IL-2 levels in peripheral blood from DTMUV-infected ducks. **a** IFN-γ levels of serum from the two groups of ducks infected with DTMUV. **b** IL-2 levels of serum from the two groups of ducks infected with DTMUV. Data were expressed as mean ± SD (n = 3). The statistical differences between both groups were evaluated by Student’s t-test. * *p <* 0.05, ** *p <* 0.01

As shown in Fig. 5a, at the early stage of DTMUV infection (3–7 dpi), increasing levels of IFN-γ in the two inoculated groups were observed. The IFN-γ levels in 15-week-old ducks were significantly higher than in 55-week-old ducks (*p <* 0.01 or *p <* 0.05). Subsequently, IFN-γ concentrations gradually declined in both groups and returned to normal levels at 13 dpi. There were significant differences for IL-2 as early as 1 and 3 dpi comparing the two groups (*p <* 0.05, Fig. 5b). At 3–7 dpi, IL-2 levels in both groups gradually rose, and peaked at 7 dpi, with a significant difference (*p <* 0.01). In the following days, the levels of IL-2 declined and returned to normal.

### Detection of neutralizing antibody level in serum

**Fig. 6:**
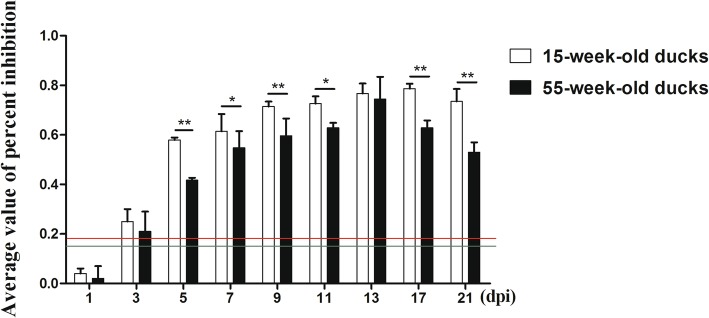
Neutralizing antibodies in the serum from DTMUV-infected ducks. Green and red lines represent critical threshold values of negative and positive antibodies, respectively. Data were expressed as mean ± SD (n = 3). The statistical differences between both groups were evaluated by Student’s t-test. * *p <* 0.05, ** *p <* 0.01

As shown in Fig. 6, positive serum could be detected in 15- and 55-week-old ducks as early as 3 dpi. However, the neutralizing antibody production in the older ducks was low and only higher than the positive control at 3 dpi. The antibody titers gradually increased in the following days, 55- and 15-week-old ducks reached the peak at 13 and 17 dpi, respectively. During the tested days, the antibody titers in the younger ducks were always higher than in the older ducks, especially in the early phase of the disease (5, 7 and 9 dpi), and there were significant differences in the two groups (*p <* 0.01 or *p <* 0.05). It should be noted that control ducks showed no sero-conversion during the experimental period.

## Discussion

Age-related outcomes of viral infections in ducks have been previously reported in highly pathogenic avian influenza virus, Japanese encephalitis virus and duck hepatitis virus [18, 20, 23]. However, few studies have focused on the pathogenicity of DTMUV in breeding ducks, so 15- and 55-week-old Cherry Valley breeding ducks were used to explore the effect of host age on the pathogenicity of DTMUV in the present study. As a result, the age-related difference in susceptibility to DTMUV in breeding ducks was significant, with the older egg-laying ducks showing more susceptibility to DTMUV than the younger reserve breeding ducks.

The 55-week-old breeding ducks infected with DTMUV showed overt clinical symptoms and gross lesions at 3–7 dpi, such as loss of appetite, depression, egg-laying drop, hemorrhage of endocardium, enlargement of liver and spleen with hemorrhage dots, as well as swelling of the lymphoid follicles of the ileum. Furthermore, the typical hyperemia, hemorrhage and deformation of the ovary, and hemorrhage of the oviduct were observed in the 55-week-old ducks, which were symptoms consistent with clinical study [1]. By contrast, the 15-week-old ducks displayed only transient symptoms and mild gross lesions, such as hemorrhage of endocardium and hepatosplenomegaly. The analysis of microscopic lesions suggested that DTMUV could cause damage to multiple tissues, mainly manifested in the hyperaemia, hemorrhage and inflammatory cell infiltration of the parenchymal organs. Interstitial hemorrhage and inflammatory cell infiltration of the heart and viral encephalitis were the most characteristic microscopic lesions in this disease, but the difference of these lesions was the varying degrees of inflammatory cell infiltration, which suggested that the immune responses induced by DTMUV were different in the two different-aged ducks [24]. Lymphocytic necrosis and a significant decrease in the number of lymphocytes were observed in the spleen, resulting in immunosuppression of infected ducks and more susceptibility to other viruses or bacteria. What was noteworthy was the severe hemorrhage and inflammatory cell infiltration in the ovary of the 55-week-old ducks, which was consistent with the egg-drop symptom. Additionally, other tissues, such as liver, kidney and lung, also showed a certain degree of pathological changes, but on the whole, the lesions of the older ducks were more severe than those of the younger ducks.

The viral loads in specific tissues are a key factor leading to tissue lesions. At 1 dpi, DTMUV could be detected in all tested tissues, indicating that DTMUV entered the body very quickly and had a broad tissue tropism. The distribution patterns of virus loads in tissues were consistent with the histopathological changes of the various tissues: the tissues where viral loads were high showed earlier and more severe lesions [22]. For example, the spleen had the highest virus loads as early as 1 dpi, and obvious lymphocyte necrosis was observed at 3 dpi in the spleen, which also indicated the spleen was the target organ of this virus [25]. However, it should be noted that the viral loads in many tissues of the 55-week-old ducks were higher than those of the 15-week-old ducks, with a significant difference.

Based on the clinical symptoms, gross and microscopic lesions as well as the viral loads in the tissues, it was believed that the 55-week-old egg-laying ducks were more susceptible to DTMUV than the 15-week-old reserve breeding ducks. However, some studies have demonstrated that the host age affected the pathogenicity of DTMUV in ducklings and goslings [21, 22, 26]. In those studies, the younger the animals were, the greater the pathogenicity that DTMUV had. What interested us was why this phenomenon happened. Recently, Lu et al. [24] investigated the effect of DTMUV infection in the different week-old breeding ducks, and also found the host age had an important role in the pathogenicity of DTMUV, especially finding that 18 to 21-week-old ducks were more susceptible to this disease, and 14 to 16-week-old ducks were more resistant, which were similar results to ours. To explain this phenomenon, we thought the immune responses were mainly responsible for this problem. The immune responses of the ducklings and the older egg-laying ducks were too weak to effectively clear the virus. Both the ovary and the spleen were the target organs, and played important roles in viral invasion and replication, DTMUV has a special affinity for these target organs, and the virus can replicate rapidly resulting in severe lesions and dysfunction.

To further describe our speculation, the levels of IFN-γ, IL-2 and neutralizing antibody in serum were measured. IFN-γ is a broad-spectrum antiviral cytokine and has function in various viral infections. IL-2 can activate T cells and promote their proliferation, leading to the production of many other cytokines involved in defending against viruses [27]. It was found that both cytokines are increased and there was a significant difference between 15-week-old ducks and 55-week-old ducks at early stages of infection, indicating that both IFN-γ and IL-2 play important roles in resistance to DTMUV infection, and the immune responses in the younger ducks were stronger than in the older ducks. Furthermore, the presence of neutralizing antibodies would help to clear the virus [28]. The changed patterns of neutralizing antibodies were similar to those cytokines, with the levels of antibodies in the older ducks significantly lower than in the younger ducks at 5–11 dpi, which also indicated that the humoral immune response to DTMUV was weaker in the 55-week-old ducks.

## Conclusions

In summary, this study demonstrated that the 55-week-old egg-laying ducks were more susceptible to DTMUV, the 15-week-old reserve breeding ducks have stronger resistance, and host age played an important role in the pathogenicity of DTMUV, this factor should be taken seriously during the prevention and control of this disease.

## Methods

### Virus

The FX2010 strain of DTMUV (accession number: KY623434.1) used in this study was isolated from the infected egg-laying ducks in Fengxian district of Shanghai in 2010 [3]. The virus was propagated in specific pathogen-free embryonated chicken eggs and the median embryo lethal dose (ELD_50_) of this virus was 10^–4.2^ ELD_50_/0.2 mL, calculated by the Reed and Muench method [29].

### Animals and experimental design

The 15- and 55-week-old ducks were purchased from Liu He Duck Farm (Shandong, China). Ducks were maintained in the animal house for two weeks before experiments. Feed and water were provided ad libitum, samples of ducks were collected to assure that the ducks were virologically and serologically DTMUV-negative using the PCR and blocking enzyme-linked immune sorbent assay (ELISA), respectively.

Eighty 15-week-old ducks were divided into two groups (40 ducks/group). Ducks of the experimental group were inoculated with 0.4 mL virus by intramuscular injection, while the age-matched control group was inoculated with 0.4 mL sterile phosphate-buffered saline (PBS) by the same method. The infected ducks were observed continuously for 21 days, and the clinical symptoms and egg production (55-week-old ducks) were recorded every day. At the indicated days (1, 3, 5, 7, 9, 11, 13, 15, 17, 19 and 21 dpi), three ducks from each group were euthanized by administration of sodium pentobarbital (100 mg/kg body weight) [30], necropsied and the gross lesions were observed. Serum samples were collected and stored at − 20 °C. The parts of tissues (heart, liver, spleen, lung, kidney, and brain) were collected and then either stored at − 80 °C for virological examinations or fixed in 10% formalin solution for histopathological examination. The remaining animals at the end of this study were also euthanized by the intravenous administration of sodium pentobarbital (100 mg/kg body weight). The experiment of 55-week-old ducks was designed as above.

### Histopathology analysis

The heart, liver, spleen, lung, kidney, and brain tissues were fixed in 10% formalin solution, embedded in paraffin, sectioned to 4 μm thicknesses and stained with haematoxylin and eosin. The stained tissue samples were observed by light microscopy for histopathological analysis.

### RNA extraction and quantitative real-time PCR

Total RNA was extracted from collected tissues using TRIzol reagent (Invitrogen, Carlsbad, CA, USA) following the manufacturer’s instructions. The concentrations of all samples were measured using an ultraviolet spectrophotometer (Shimadzu, Shimazu, Japan), and cDNA was synthesized from 1 μg total RNA using PrimeScript TM RT Reagent Kit (TaKaRa, Dalian, China) with a gDNA Eraser Kit (TaKaRa, Dalian, China). qPCR primers of viral E gene (GenBank No.: HQ833330) were designed and the standard curve was established based on the previous report, the forward primer was: 5′-CGCTGAGATGGAGGATTATGG-3′; the reverse primer was: 5′-ACTGATTGTTTGGTGGCGTG-3′, and the positive product of qPCR was 225 bp [31]. To confirm the viral copy number in the infected ducks, values were normalized per 1 μg total RNA. qPCR was performed in the Applied Biosystems 7500 Fast Real-Time PCR System (Applied Biosystems, Foster City, CA, USA), and the total volume was 20 μL, including 2 μL of cDNA as template, 10 μL of 2 × TransStart Tip Green qPCR SuperMix, 0.4 μL of 50 × Reference Dye RoxII, 0.4 μL each of primers (10 μM) and 6.8 μL of RNase free H_2_O. qPCR conditions consisted of 94 °C for 30 s, 45 cycles of amplification at 94 °C for 5 s, and 60 °C for 34 s, followed by analysis by melt curve. Each sample was analyzed in triplicate.

### Detection of IFN-γ, IL-2, and neutralizing antibody in serum

The concentrations of serum IFN-γ and IL-2 were determined using the ELISA Kit (Langton, Shanghai, China) following the manufacturer’s instructions. The neutralizing antibody titers were measured by a blocking ELISA [8]. Briefly, ELISA plates were coated with about 3 μg/well purified FX2010 strain in 0.1 M carbonate–bicarbonate buffer (pH 9.6) and incubated overnight at 4 °C. The coated plates were washed with PBS (pH 7.4) containing 0.05% Tween-20 (PBST), and 100 μL of blocking buffer (PBS containing 5% skim milk) was added to block the nonspecific binding sites for 1 h at 37 °C. Serum samples were diluted and added to each well and incubated for 1 h at 37 °C. The wells were incubated with monoclonal antibody 1F5 (20×) for 1 h at 37 °C and afterwards washed with PBST three times. Then wells were washed three times with PBST and incubated with a second antibody (goat anti-mouse IgG conjugated to HRP) at room temperature for 1 h. After the wells were rinsed with PBST three times, 100 μL of 3,3′,5,5′-tetramethyl benzidine was added into the wells for incubation 5 min at room temperature. The reaction was stopped by 0.1 N sulfuric acid. The optical density (OD) was measured at 450 nm, and the percent inhibition (PI) value was determined using the formula: PI (%) = (1 - (OD450_nm_ of test serum/OD450_nm_ of negative control serum)) × 100%. Diploid positive and negative controls were used during this test.

### Statistical analysis

All data were expressed as means ± standard deviation (SD), and an independent-sample *t*-test was used to evaluate data using Graph Prism software (GraphPad Software, San Diego, CA, USA). The statistical significance was set at *p <* 0.05, indicated by an asterisk (*), and the extremely significant difference was set at *p <* 0.01, indicated by a double asterisk (**).

## Data Availability

The data involving in the manuscript can be obtained from the corresponding author upon reasonable request.

## Abbreviations

dpi: Days post infection
DTMUV: Duck Tembusu virus
ELD_50_: Median embryo lethal dose
ELISA: Enzyme-linked immune sorbent assay
IFN-γ: Interferon gamma
IL-2: Interleukin 2
PBS: Phosphate-buffered saline
qPCR: Quantitative real-time polymerase chain reaction
